# Opalski Syndrome and Elucidation of Lateral Medullary Syndrome

**DOI:** 10.7759/cureus.54314

**Published:** 2024-02-16

**Authors:** Anthony T Joseph, Ava Toluie, Peter A Hrehorovich

**Affiliations:** 1 Orthopedic Surgery, Lake Erie College of Osteopathic Medicine, Bradenton, USA; 2 Pediatrics, Lake Erie College of Osteopathic Medicine, Bradenton, USA; 3 Diagnostic Radiology, Advent Health Sebring, Sebring, USA

**Keywords:** radiology, neurology, stroke, vertebral artery, pica, lms, posterior inferior cerebellar artery, posterior circulation stroke, opalski syndrome, lateral medullary syndrome (wallenberg syndrome)

## Abstract

In this case, a 61-year-old patient presented with Horner’s syndrome of the left eye, left-sided truncal ataxia, left-sided pain/paresthesia of the face as well as right-sided loss of pain and temperature of the arms and legs. MRI findings displayed a clear 4 mm acute left lateral medullary infarct of the left posterior inferior cerebellar artery (PICA) vascular territory, indicative of lateral medullary syndrome (LMS). The presence of pre-existing medical conditions such as uncontrolled diabetes, late-stage syphilis, and a mechanical aortic valve complicated this clinical picture. The presence of ipsilateral corticospinal deficits in this patient revealed Opalski syndrome, a rare variant of LMS. This case report highlighted the importance of correlating imaging and physical examination of stroke findings.

## Introduction

Cerebrovascular disease constitutes a substantial contributor to disability and mortality in the United States. The primary etiology of cerebrovascular accidents can be attributed to ischemic events, which account for approximately 85% of cases, with the remaining 15% arising from hemorrhagic origins [1]. Cerebrovascular accidents often involve the circle of Willis, a vascular plexus formed by the anterior internal carotid artery (ICA) and posterior cerebral (vertebral) vasculature. Within the posterior cerebral vasculature, occlusion or hemorrhage affecting the vertebral artery (VA) or the posterior inferior cerebellar artery (PICA) may give rise to the clinical syndrome known as lateral medullary syndrome (LMS) or Wallenberg syndrome [2].

LMS is classically characterized by a constellation of neurological findings, which include ipsilateral Horner's syndrome, ipsilateral vestibulocerebellar symptoms, ipsilateral bulbar weakness, and crossed sensory symptoms [3]. Therefore, a patient presenting with LMS typically exhibits a loss of pain and temperature sensation on the ipsilateral face and contralateral body. Additionally, they may display ipsilateral cerebellar ataxia, leading to a propensity to fall toward the side of the lesion, and ipsilateral dysarthria and dysphagia, often manifesting as a loss of the gag reflex on the same side [2,4].

The clinical manifestations of LMS have been found to vary and when hemiplegia or hemiparesis is a consequence, two syndromes must be considered: Opalski syndrome and Babinski-Nageotte syndrome. Opalski syndrome has ipsilateral disease manifestations, whereas Babinski-Nageotte syndrome has contralateral deficits.

## Case presentation

A 61-year-old patient presented to the emergency department (ED) with left-sided paresthesias and hemiparesis of the arm and leg. The patient reported that they awoke from sleep due to left-sided weakness with difficulty moving the left leg, diminished sensation, and ataxic gait which resulted in multiple near falls since they would repeatedly lean to the left side. The patient also described left craniofacial pain. The patient had a past medical history of hypertension, hypercholesterolemia, uncontrolled diabetes mellitus type two (T2DM), chronic kidney disease 3a, human immunodeficiency virus, late-stage syphilis, coronary artery disease status post coronary artery bypass graft times three, and aortic stenosis status post a mechanical aortic valve. 

On examination, the patient had difficulty lifting the left arm, a left Horner’s pupil with mild ptosis and miosis, loss of temperature sensation on the right side of the body, and numbness on the left side of the face and right side of the body. The patient had a negative skew test and no swelling or trauma to the extremities. Deep tendon reflexes were absent in the lower extremities. NIH stroke score (NIHSS) upon presentation was determined to be a three (minor severity) based on left upper extremity motor drift and sensory deficit, and blurred vision (diplopia). 

**Figure 1:**
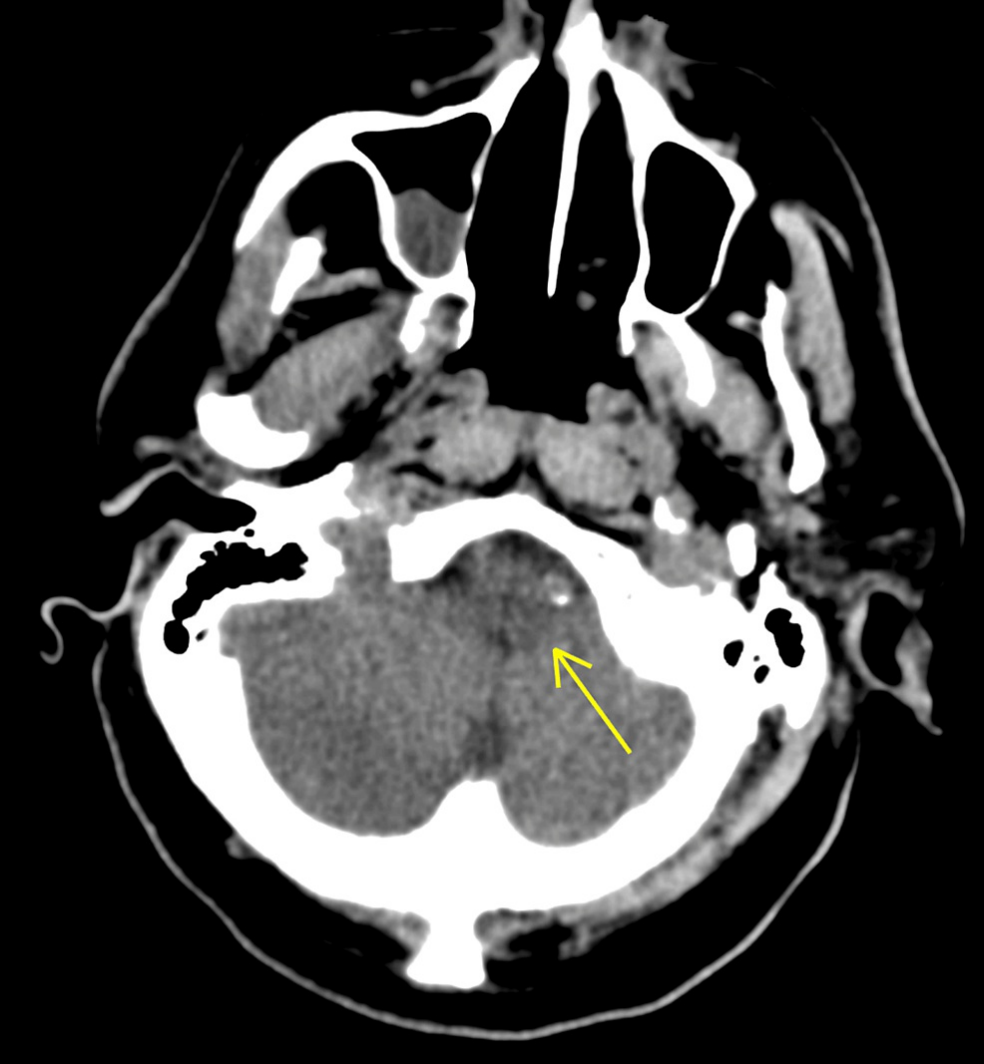
Head CT demonstrating no acute intracranial abnormality.

**Figure 2:**
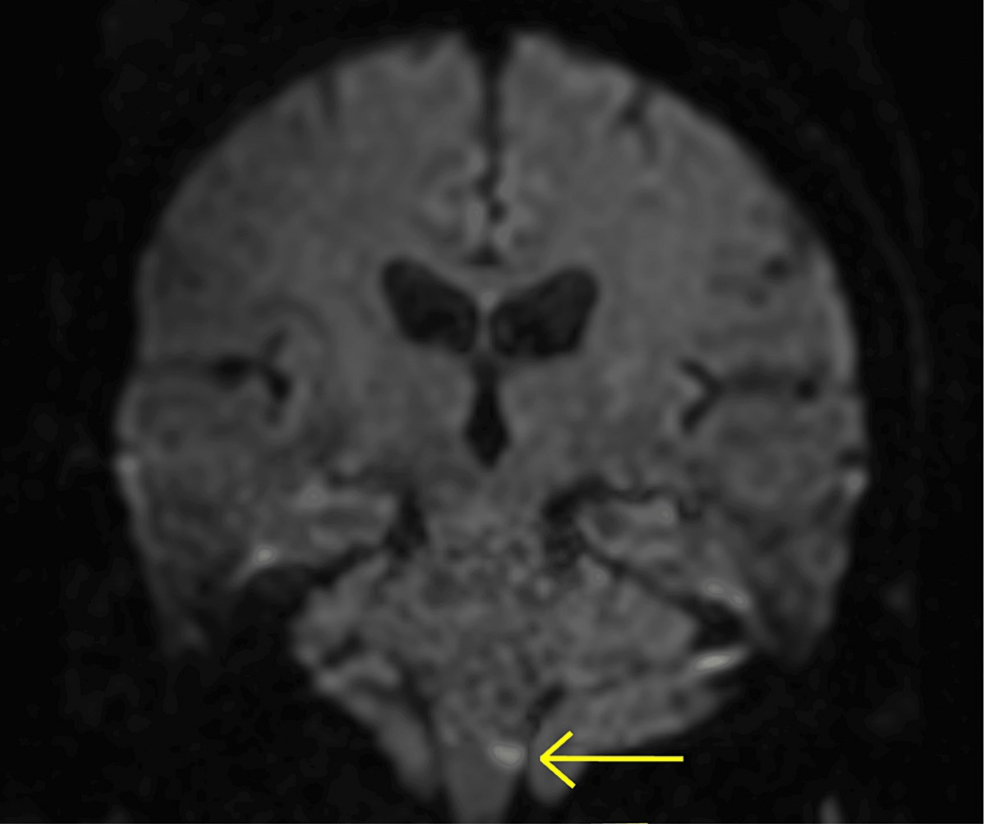
Coronal diffusion-weighted imaging displays the 4 mm hyperintense acute infarct of the left medulla.

**Figure 3:**
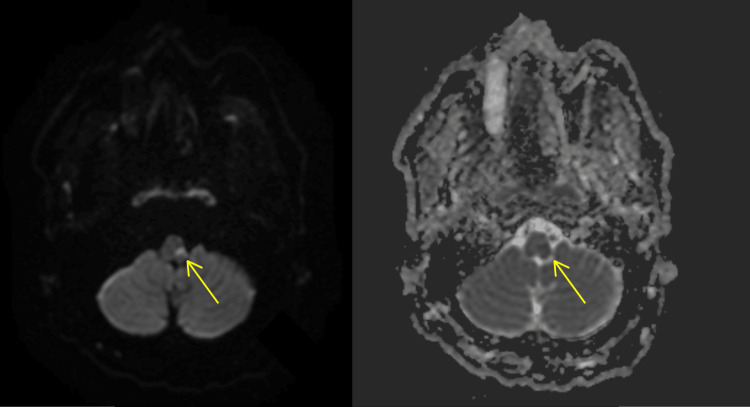
Axial diffusion-weighted imaging of the middle medulla demonstrating the 4 mm acute infarct of the left medulla (Left). Apparent diffusion coefficient map reveals corresponding low signal alteration (Right).

The patient was not administered tissue plasminogen activator as it had been more than four and half hours since their symptoms began. Rather, secondary stroke prevention was administered with low-dose aspirin 81 mg QD and atorvastatin 80 mg QD. The patient was not treated with dual antiplatelet therapy as they were already anticoagulated with a therapeutic dose of warfarin (INR = 2.9), since they had a mechanical aortic valve. Adding another antiplatelet would have risked the conversion of the patient's thromboembolic stroke into a hemorrhagic stroke. Computed tomography (CT) of the head without contrast revealed no acute intracranial abnormality (Figure 1). A carotid ultrasound displayed moderate stenosis of the right internal carotid artery (ICA). A CT angiography of the head and neck showed no large vessel disease in the head or intracranial stenosis, but confirmed 50% stenosis of the right ICA. Due to persistent left-sided weakness, a comparison non-contrast CT of the head was performed and revealed a subtle diffuse hypodensity throughout the right frontoparietal temporal region. These findings were concerning for an evolving right middle cerebral artery (MCA) infarct however, magnetic resonance imaging (MRI) confirmed no infarct. Furthermore, an MRI of the brain without contrast revealed a 4 mm acute left lateral medullary infarct of the left posterior inferior cerebellar artery (PICA) vascular territory (Figures 2, 3). There was also mild diffuse central and cortical atrophy with mild chronic ischemic small-vessel disease.

The patient’s left-sided mobility and weakness had improved to a three to four out of five strength three days after admission. The patient was discharged home after four days with warfarin 7.5 mg QD, and meclizine for their dizziness, as well as recommendations to proceed with physical and occupational therapy with a front wheel walker. 

## Discussion

Wallenberg syndrome or LMS can be demonstrated through clear neurological deficits associated with the neuroanatomy affected by malperfusion of the vertebral artery or its extension, PICA. As part of the posterior cerebral vasculature, obstruction of the vertebral artery is a more common cause of LMS in comparison to obstruction of the PICA. Approximately 67% of lateral medullary strokes are associated with the vertebral artery as opposed to 10% for the PICA [5]. The development of LMS is a consequence of large-vessel infarction in 50% of cases while cardioembolism is the culprit 5% of the time. Additionally, 15% of LMS cases are from arterial dissections and 13% are from small vessel infarcts [2,6]. This case is unique due to the pathophysiology behind the occurrence of the stroke and the vascular structure affected (PICA). The patient's past cardiac medical history of a mechanical aortic valve and atherosclerosis suggested that the stroke could be either ischemic or thromboembolic in origin. Therefore, the stroke's origins are dualfold as the patient was already at high risk. 

Concerning the physical exam findings seen in this patient, the presence of corticospinal deficits called for further classification between Opalski syndrome and Babinski-Nageotte syndrome. In Opalski syndrome, there are ipsilateral symptoms due to the infarct extending caudally to include the fibers of the lateral corticospinal tract after the pyramidal decussation [7-9]. Contralateral hemiparesis occurs in Babinski-Nageotte syndrome because the lateral corticospinal tract is affected before it crosses at the pyramidal decussation [7]. This case report elucidates a clear picture of LMS on MRI, in addition to examination findings that are unique to Opalski syndrome. Similar cases have been reported demonstrating ipsilateral corticospinal deficits in the context of LMS where there is a positive Babinski sign as well as contralateral sensory deficits [7,9,10].

Traditionally, LMS is associated with ipsilateral trigeminal and contralateral spinothalamic sensory loss. In this patient, the presence of areflexia and sensory loss of the lower extremities suggests peripheral neuropathy due to uncontrolled T2DM complicating a clear picture of the acute stroke deficits [11]. Additionally, while diplopia can be a sequelae of LMS, due to damage to the otolithic vestibular nuclei [12], this patient had a negative skew test and their diplopia was self-reported by the patient to have existed before the stroke. The absence of lesions in the pons or midbrain also supported the conclusion that this patient's diplopia was not from their stroke. 

In patients with LMS, physiotherapy, antithrombotic, and statin therapy are the cornerstone of treatment. In addition, control of modifiable risk factors such as hypertension, diabetes, and smoking are also of utmost importance. Although this patient did not present with dysphagia, a common deficit involved in LMS, patients with dysphagia may benefit from visits with a speech therapist and nutritionist to help guide treatment [4].

## Conclusions

Lateral medullary syndrome is the most common ischemic stroke presentation associated with the posterior cerebral vascular anatomy. While various studies have demonstrated the typical deficits associated with LMS, reports that show deviation from these findings in combination with imaging allow for a proper diagnosis. In this case report, the presence of preexisting medical conditions and ipsilateral corticospinal deficits paint a unique picture of the stroke’s etiology, evolution, and resultant clinical manifestations. Lastly, highlighting Opalski syndrome encourages healthcare professionals to be mindful of its presence when faced with patients who had an LMS stroke.
